# Redox-sensitive lipophilic prodrugs: delivering unstable chemotherapeutant for improved cancer therapy

**DOI:** 10.1080/10717544.2019.1678696

**Published:** 2019-11-18

**Authors:** Fu Li, Zhao Huang, Huitong Chen, Lu Yan, Jin Li, Yue Su, Qian Zhang, Zhengye Huang, Yaxin Zheng

**Affiliations:** School of Pharmacy, Key Laboratory of Sichuan Province for Specific Structure of Small Molecule Drugs, Chengdu Medical College, Chengdu, China

**Keywords:** Camptothecin, lipophilic prodrugs, antitumor activity, drug delivery, redox sensitivity

## Abstract

Therapeutic application of unmodified camptothecin (CPT) is severely restricted by its extremely low water solubility and the instability of active lactone ring. In this study, a redox-sensitive CPT-OA conjugate containing the disulfide bond (CPT-SS-OA) was used to deliver the lactone-stabilized CPT for the improved antitumor efficacy. A non-sensitive CPT-OA was used as control to illuminate the role of disulfide bond. Both CPT-SS-OA and CPT-OA formulated in cremophor EL micelles (CM) displayed multiple therapeutic advantages: small diameter (∼14 nm), efficient cellular internalization, prolonged blood circulation, and favorable biodistribution. However, only CPT-SS-OA/CM achieved the superior chemotherapeutic efficacy over CPT solution in the Lewis lung carcinoma (LLC) cancer xenograft, which was ascribed to the accelerated release of the active lactone CPT responding to the elevated reductive glutathione in tumor cells. Such redox-sensitive lipophilic prodrugs represent an effective alternative strategy for the delivery of CPT in the active lactone form. This strategy can be used for other chemically unstable chemotherapeutant for the improved therapeutic efficacies.

## 1. Introduction

Camptothecin (CPT) is a potent antitumor agent against various cancers through the pathway of topoisomerase-1 inhibition (Martino et al., 2017). However, CPT cannot be directly administered due to its very low solubility in water. Various strategies attempted to enhance the aqueous solubility of CPT through the physical entrapment in nanocarriers only meet with limited success (Kunii et al., 2007; Liu et al., 2009; Ueki et al., 2009; Jang et al., 2016), which is mainly ascribed to the high crystallization ability of CPT as a result of the planar chemical structure. Moreover, CPT is extremely insoluble in organic solvents (except for dimethyl sulfoxide), which makes it hard to be formulated into the frequently-used nanocarriers (Botella & Rivero-Buceta, 2017). In addition to its aqueous insolubility, the active closed-ring CPT (lactone) can be readily converted to the inactive open-ring CPT (carboxylate) under physiologic condition. After intravenous injection, the carboxylate form of CPT can preferentially bind to albumins, thus driving the lactone-carboxylate equilibrium toward the formation of inactive open-ring CPT (Martino et al., 2017; Wen et al., 2017). Such carboxylate CPT is mainly cleared by the kidneys and causes hemorrhagic cystitis and myelotoxicity, which caused the suspension of the clinical trials (Thomas et al., 2004). To address these challenges, strategies to overcome the aqueous insolubility and poor chemical instability of unmodified CPT remain a research hotspot in the recent scientific literatures (Alibolandi et al., 2017; Tian et al., 2017; Zhan et al., 2017; Li et al., 2018a; Dong et al., 2019; Zhang et al., 2019).

Small-molecule amphiphilic prodrugs have been widely to overcome the limitation of the poor aqueous solubility of hydrophobic antitumor drugs (Ma et al., 2017). They are usually synthesized by conjugating hydrophobic drugs to a hydrophilic segment, such as hydrophilic drugs, peptides, and polyethylene glycol. Due to their amphiphilic nature, these amphiphiles can readily self-assemble into nanoaggregates (NAs), which show many advantages (e.g. precisely defined chemical structure and high loading capacity) as compared to their polymeric counterparts (Cheetham et al., 2017). However, such hydrophilic modification also results in a decreased thermodynamic stability of NAs, which leads to a high risk of NAs disassembly and subsequently burst drug release in bloodstream after intravenous injection (Lin & Cui, 2015).

On the contrary, NAs based on the lipophilic prodrugs of hydrophobic drug (LHPs) show the high thermodynamic stability due to its increased hydrophobic nature (Li et al., 2019). Although it is commonly observed that highly hydrophobic molecules alone form large precipitations when dispersing in water, LHPs have been recently demonstrated to self-assemble into stable NAs without the presence of stabilizing agents (Dosio et al., 2010; Wang et al., 2014; Li et al., 2018a). However, LHP-NAs usually form large precipitates in the phosphate-buffered saline (PBS) or plasma, probably due to the collapse of the effect of electrostatic stabilization (Zheng et al., 2019). Therefore, PEGylation of LHP-NAs (usually with DSPE-mPEG_2000_ and cremophor EL) is required for their therapeutic applications for the efficient delivery of hydrophobic drugs. To date, LHP-NAs have been widely used for the delivery of various hydrophobic drugs (e.g. paclitaxel, SN38, docetaxel, curcumin, and doxorubicin) with improved antitumor efficiencies (Maksimenko et al., 2014; Wang et al., 2014; Cheikh-Ali et al., 2015; Zhang et al., 2017a; Zheng et al., 2019).

Inspired by these findings, we speculated that LHP-NAs could act a promising tool to delivery CPT with the active lactone structure protected, because that (1) the chemical modification at the hydroxy of CPT could stabilize the lactone ring of CPT prodrugs (Cao et al., 2009; Dong et al., 2019). (2) LHPs in the aggregated stage can hinder the premature release of CPT by decreasing the number of prodrug molecules available for enzyme-mediated hydrolysis (Li et al., 2019). (3) LHPs readily enter the cytoplasm through a direct transmembrane transportation by inserting themselves into cell membrane (Zeng et al., 2019). (4) reduction-responsive linkers (i.e. disulfide bond) can be introduced to achieve the selective release upon the high reductive environment in tumor cells (Luo et al., 2016; He et al., 2017; Zhang et al., 2017b; Yu et al., 2018; He et al., 2019), which are characterized as elevated intracellular glutathione (GSH) (He et al., 2012).

In this study, we investigate whether the redox-responsive LHPs could effectively delivery the lactone-stabilized CPT for the improved cancer therapy, with emphasis to evaluate the role of disulfide bond. Therefore, two types of LHP were synthesized: one with a reduction-responsive disulfide bond and the other with a non-responsive linkage as control. Although redox-sensitive LHPs have been used for the delivery of CPT with the potent *in vivo* antitumor efficacies (Lu et al., 2015; Li et al., 2018a), the remarkably different physicochemical properties (i.e. sizes, zeta potentials, and morphologies) between redox-responsive NAs and non-responsive NAs made it difficult to make an explicit conclusion. To address this, the two lipophilic CPT prodrugs were formulated in cremophor EL micelles (CM) with the same physicochemical properties, which were then compared with respect to the reduction-triggered drug release, *in vitro* cytotoxicity, biodistribution and *in vivo* antitumor activity.

## 2. Materials and methods

### 2.1. Materials and animals

Oleic acid (OA) was obtained from Sigma-Aldrich. 2,2-Dithiodiethanol, hexanediol, and dicyclohexylcarbodiimide (DCC) were purchased from Alfa Aesar (MA). 4-dimethylaminopyridine (DMAP) and triphosgene were purchased from Adamas Reagent (China). Curcumin (CUR), CPT, DiR, Dithiothreitol (DTT), LysoTracker and AannexinV-FITC apoptosis detection kit were purchased from MEILUN Biology Technology Co., Ltd. (Dalian, China). The DSPE-mPEG_2000_ was purchased from Lipoid GmbH (Ludwigshafen, Germany). Cremophor EL was obtained from BASF (Ludwigshafen, Germany). All solvents used in this study were analytical grade. Male C57 mice (6–8 weeks old) and Sprague–Dawley rats (200–250 g) were purchased from the Laboratory Animal Center of Chengdu Medical college (Chengdu, China). All studies involving mice were approved by the institute’s animal care and use committee.

### 2.2. Synthesis of CPT-SS-OA and CPT-OA

**Scheme 1.**
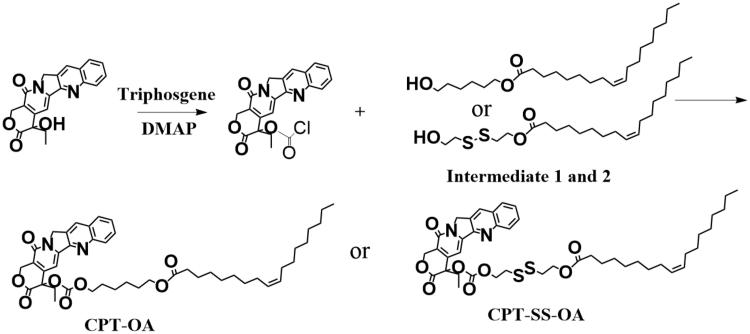
Synthesis of CPT-OA and CPT-SS-OA.

Scheme 1 shows the synthesis route of CPT-SS-OA and CPT-OA. Intermediates 1 and 2 were synthesized by coupling OA to 2,2-dithiodiethanol or hexanediol with the catalysis of DCC and DMAP in dichloromethane (DCM) (Zheng et al., 2019). CPT was then conjugated to the intermediates using the previously reported method (Lu et al., 2015). Briefly, CPT (100 mg) and DMAP (2.0 molar eq.) was dissolved in 20 ml anhydrous DCM at 0 °C under nitrogen. Triphosgene (0.35 molar eq.) in the anhydrous DCM was then added dropwise, and the solution was stirred at 0 °C for 20 min. The intermediate (1.0 molar equiv.) was then added, and reaction was performed at room temperature overnight. The solution was dried and purified on the silica gel plate chromatography (Methanol/DCM, 1:20) to obtain the corresponding CPT-SS-OA and CPT-OA (yield, ∼50%).

### 2.3. Preparation and characterization of CPT-SS-OA/CM and CPT-OA/CM

CPT-SS-OA/CM and CPT-OA/CM were prepared by diluting the CPT-SS-OA and CPT-OA solution (10 mg/ml) in ethanol/Cremophor EL (5/1, v/v) in saline to reach a desired concentration, respectively. The size, size distribution and zeta potential of CMs were measured using Zetasizer (Nano-ZS90, Malvern, England). Transmission electron microscopy (TEM, JEM-1200EX, Japan) was utilized to observe the morphology of CMs, samples were stained with 2% uranyl acetate. CPT solution was prepared by dissolving 10 mg of CPT in 0.1 M NaOH, and the pH of solution was adjusted to 7.4 with 1 M HCl.

### 2.4. Evaluation of CPT release using FRET

CPT release from lipophilic prodrugs was evaluated using a fluorescence resonance energy transfer (FRET) technique as described before (Li et al. 2018b). Briefly, a non-sensitive lipophilic prodrug of curcumin (CUR-OA) was chosen as the FRET acceptor, with CPT-OA or CPT-SS-OA as the FRET donor. CUR-OA and CPT-OA (or CPT-SS-OA) were co-loaded in the CM to obtain the CPT-CUR/CM or CPT-SS-CUR/CM. CPT-CUR/CM or CPT-SS-CUR/CM were then supplemented with 1 ml of 10 mM phosphate buffer (PB, pH 7.4) containing 0 and 10 mM DTT, respectively. The solution was incubated at 37.5 °C, CPT fluorescence was determined by a fluorescence spectrometer at the given time interval (λ_ex_ = 362 nm and λ_em_ = 426 nm). CPT fluorescence would be readily activated due to the release of CPT from CPT-CUR/CM, which could be used to evaluate CPT release from lipophilic prodrugs.

### 2.5. *In vitro* hydrolysis profiles of CPT from prodrugs

CPT-SS-OA/CM and CPT-OA/CM were supplemented with 1 ml of 10 mM PB (pH 7.4) containing 10 mM DTT at a final CPT equivalent concentration of 10 μg/ml in the sample vials. The vials were then incubated in a water bath at 37 °C, and 20 μl solution was withdrawn for HPLC analysis (1260 Infinity, Agilent) at the given time interval.

### 2.6. Cellular uptake

Lewis lung carcinoma (LLC) cells were purchased from American Type Culture Collection (Rockville, MD). LLC cells were maintained in DMEM containing 10% FBS, penicillin (100 units/ml) and streptomycin (100 μg/ml) in a humidified atmosphere of 5% CO_2_ at 37 °C. LLC cells were exposed to the CPT-SS-OA/CM and CPT-OA/CM and consequently incubated at an equivalent CPT concentration of 5 μg/ml for 2 h at 37 °C, respectively. Cells were washed three times with cold PBS and fixed with 4% paraformaldehyde for 30 min. The cells were further incubated with LysoTracker at a concentration of 50 nM for 1.5 h to label lysosomes. The images of cells were observed using a fluorescence microscopy (Olympus, BX63).

### 2.7. Cytotoxicity assay

The cell viability was assessed by MTT assay. Briefly, LLC cells were seeded in a 96-well plate at a density of approximate 5000 cells per well. After 24 h of growth, the medium was changed with the medium that contained CPT-SS-OA/CM, CPT-OA/CM and free CPT at various concentrations. The cell was further incubated for 48 h, and these without any treatment were utilized as control.

### 2.8. Cell apoptosis analysis

LLC cells in a 12-well plate (1 × 10^5^ cell/well) were incubated with CPT-SS-OA/CM, CPT-OA/CM and free CPT at an equivalent CPT doses of 1 μg/ml for 48 h at 37 °C. The cells were then collected consecutively by trypsinization and centrifugation and washed with cold PBS for several times. The cells were stained with annexin V-FITC and propidium iodide (PI) using the AannexinV-FITC apoptosis detection kit, and apoptosis analysis was performed on a flow cytometer (NovoCyte 3130, ACEA).

### 2.9. Pharmacokinetics

Sprague–Dawley rats (200–250 g) were used for the pharmacokinetics studies. Male Sprague-Dawley rats (4 rats/per group) were assigned to one of three groups, which received an intravenous bolus of CPT-SS-OA/CM, CPT-OA/CM and CPT solution at an equivalent CPT dose of 3 mg/kg. At the indicated time points, about 0.5 ml blood samples were taken and centrifuged to obtain the plasma sample. The concentration of CPT-SS-OA, CPT-OA/CM and CPT in plasma was analyzed by a validated HPLC method.

### 2.10. Biodistribution

*Ex vivo* imaging was performed in the colorectal cancer cells (CT26) tumor-bearing BALB/C mice (tumor volume, around 500 mm^3^) receiving the intravenous injection of DiR solution, CPT-OA/CM and CPT-SS-OA/CM (labeled with DiR) at an equivalent CPT dose of 10 mg/kg (DiR, 0.1 mg/kg). At the indicated times (12, 24, and 48 h), the mice were sacrificed, and the tumor, spleen, heart, lungs, liver, and kidneys were excised and imaged using an IVIS Lumina III living image system (PerkinElmer) with the excitation at 730 nm and emission at 790 nm. The mean fluorescence intensity of each organ was determined and compared.

### 2.11. *In vivo* antitumor efficacy

The antitumor efficacy of CPT-SS-OA/CM and CPT-OA/CM were investigated in LLC tumor-bearing mice. A subcutaneous model of lung cancer was established by subcutaneously inoculating LLC cells (2 × 10^6^ cells per 100 μl) into the right axillary flank region of male C57 mice. When tumors in the mice reached a volume of 50–100 mm^3^, the mice were randomly assigned to four groups (*n* = 6) and intravenously injected with multiple injections (every 3 days × 3) of saline, CPT-SS-OA/CM, CPT-OA/CM, and CPT solution at an equivalent CPT dose of 10 mg/kg. Animal weight and tumor volume were measured every 2 days, and tumor volume was calculated using the formula (*L* × *W*^2^)/2, where *L* is the longest and *W* is the shortest diameter tumor. All the mice were sacrificed six days post the final injection, and all tumors were harvested and weighed. The aspartate transaminase (AST), alanine transaminase (ALT), blood urea nitrogen (BUN), and creatinine were assayed to evaluate the hepatic and renal function. The major organs were collected and stained by H&E to identify the pathological change.

### 2.12. Statistical analysis

All acquired data are presented as an average value and its standard deviation, shown as the mean ± SD. Student’s *t*-test and one-way analysis of variance (ANOVA) were utilized to analyze the differences in the groups; *p* < .05 was considered statistically significant.

## 3. Results and discussion

### 3.1. Preparation and characterization of CPT-SS-OA/CM and CPT-OA/CM

**Figure 1.**
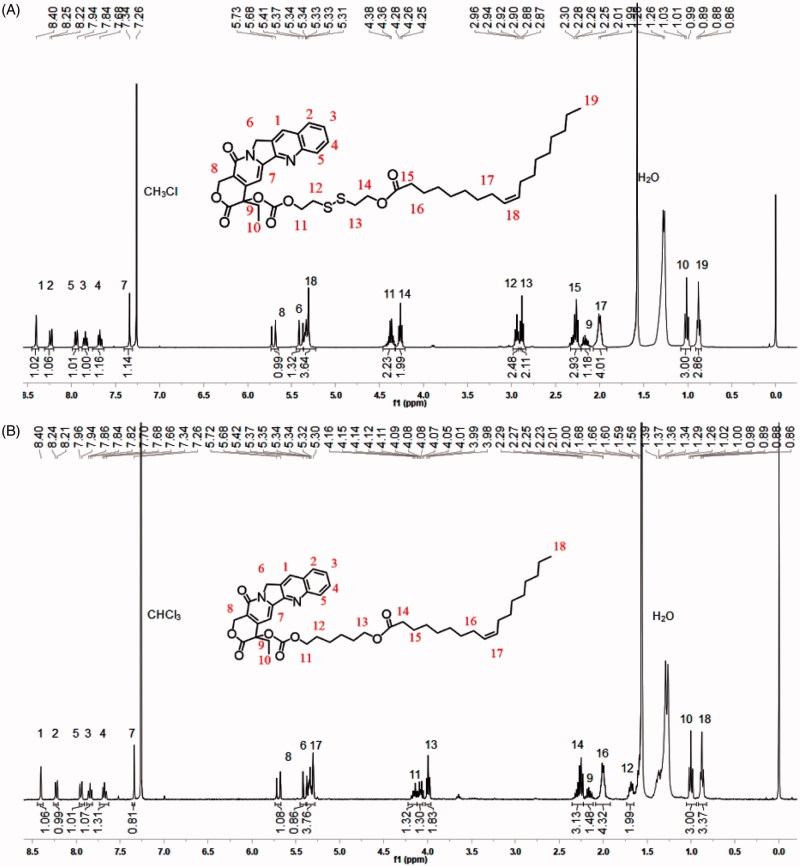
^1^H-NMR of CPT-SS-OA(A) and CPT-OA (B).

The lipophilic prodrug of CPT with the disulfide bond (CPT-SS-OA) was synthesized by conjugating OA to CPT via the linker of disulfanyl-ethyl carbonate (Li et al., 2018a). The nonsensitive CPT prodrug (CPT-OA) was synthesized by coupling OA to CPT via the hexyl carbonate linker. Their chemical structures were confirmed by the ^1^H-NMR (Figure 1).

**Figure 2.**
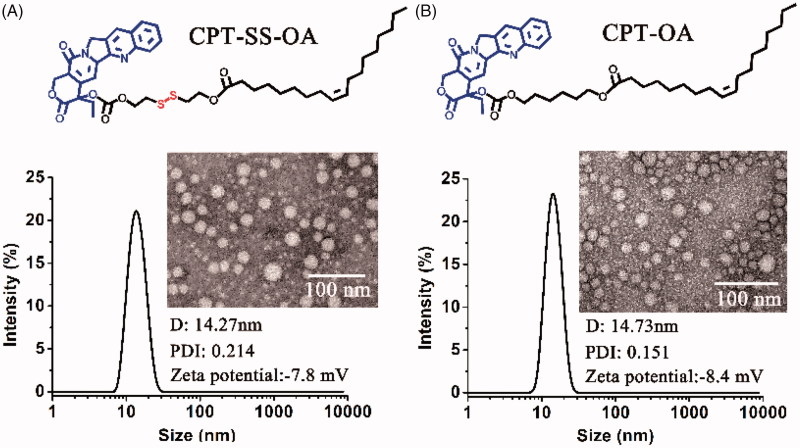
Size distribution, zeta potential and TEM image of CPT-SS-OA/CM (A) and CPT-OA/CM (B).

Both CPT-OA and CPT-SS-OA were formulated in CM by diluting the CPT prodrugs solutions (ethanol/Cremophor EL, 5/1) in the saline. As shown in Figure 2, both CPT-OA/CM and CPT-SS-OA/CM show the nearly same average hydrodynamic diameter (∼14 nm) with the similar size distributions, zeta potential and spherical morphology. The similar particle sizes were also observed between the prodrug-loaded CM and blank CM (13.3 nm), indicating that lipophilic CPT prodrugs were encapsulated in the hydrocarbon palisade region of CM without the significant influence on the properties of CM (Zeng et al., 2019). Such identical properties of CPT-OA/CM and CPT-SS-OA/CM would favor the comparative study to illuminate the role of the reduction-responsive linker (disulfide bond) on the delivery of lactone-stabilized CPT, without the interference from different physicochemical properties.

Although DSPE-mPEG_2000_ was widely used for the PEGylation of LHP-NAs (Wang et al., 2014; Li et al., 2018a; Zheng et al., 2019), their morphologies and particle sizes were highly dependent on the chemical structure of LHPs (Li et al., 2018a). CrEL has also been demonstrated to be more advantageous than DSPE-mPEG_2000_ in terms of the improvement of antitumor activity (Zeng et al., 2019). Furthermore, the CM with such small particle sizes were supposed to be suitable for the enhanced tumor penetration, as the small nanoparticles have been demonstrated to penetrate the tumor more readily than those with larger particle sizes (Cabral et al., 2011; Wang et al., 2015).Therefore, CrEL rather than DSPE-mPEG_2000_ was used for the PEGylation of CPT-OA and CPT-SS-OA for comparative studies. It is worth mentioning that the quantity of CrEL used for preparation of CM was only one fifth of that for Taxol, which could potentially decrease the hypersensitivity reaction caused by CrEL (Gelderblom et al., 2001). The reason why LHPs could be readily encapsulated in CM was the co-presence of long alkyl chain in LHPs and Cremophor EL. Therefore, other amphiphiles containing long alkyl chain, like Tween 80, might be also used for the formulation of CPT-SS-OA and CPT-OA.

### 3.2. CPT release triggered by reductive stimulus

**Figure 3.**
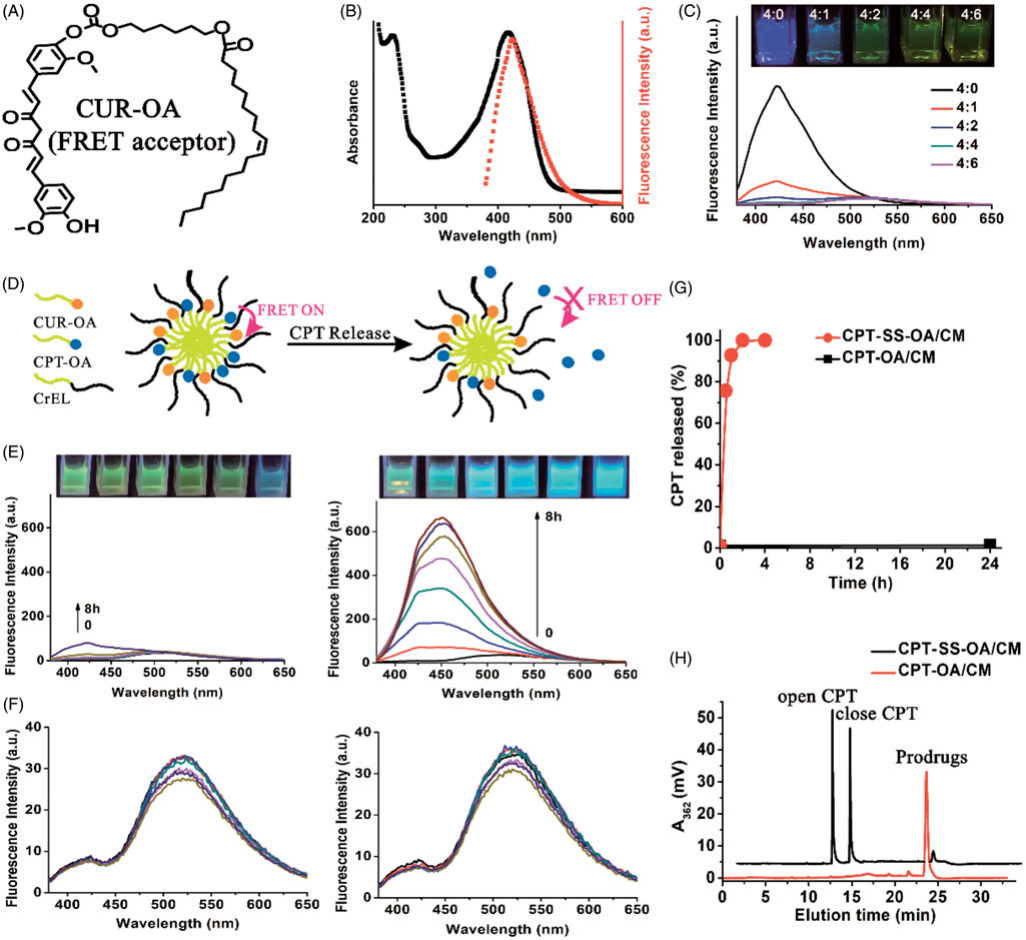
Chemical structure of CUR-OA as a FRET acceptor (A). The emission spectrum of CPT-OA/CM overlapped with the absorption spectrum of CUR-OA/CM, indicating a good FRET pair (B). Effect of CPT-OA/CUR-OA ratio (mole/mole) on the quenching in CPT fluorescence of CPT-CUR/CM (C). Schematic illustration of the FRET CPT-CUR/CM to monitor CPT release (D). The kinetic changes of appearance under a UV lamp with excitation at 365 nm and emission spectra in 10 mM PB (pH 7.4) containing 10 mM DTT (E) and 0 mM DTT (F). CPT release from CPT-OA/CM and CPT-SS-OA/CM in 10 mM PB containing 10 mM DTT (G). Typical HPLC analysis of CPT-OA/CM (24 h) and CPT-SS-OA/CM (1 h) in 10 mM DTT (H).

CPT release was investigated using a FRET technique according to previous reports (Li et al., 2018b). The non-sensitive CUR-OA conjugate was synthesized and used as the FRET acceptor (Figure 3(A)), with the CPT-OA or CPT-SS-OA itself used as FRET donor. As shown in Figure 3(B), the emission spectrum of CPT-OA/CM overlapped well with the absorption spectrum of CPT-SS-OA/CM, demonstrating a good FRET pair. The CPT-OA and CUR-OA were then co-encapsulated into CM, of which CPT fluorescence was gradually quenched with the increase of amount of CUR-OA (Figure 3(C)). This result indicated that lipophilic prodrugs of CPT and CUR could be stably co-entrapped in CM, resulting in the quenching of CPT fluorescence. To guarantee the sufficient quenching of CPT fluorescence, the CPT-CUR/CM at the CPT-OA/CUR-OA ratio of 1/1.5 (mole/mole) were used in the following studies.

As shown in the schematic illustration (Figure 3(D)), CPT fluorescence would be activated once CPT was released, because that CPT itself could not be readily entrapped in CM. Therefore, CPT release could be readily monitored by detecting the kinetic change of CPT fluorescence in a visualized manner. The CPT-CUR/CM and CPT-SS-CUR/CM then incubated at 37.5 °C in the 10 mM PB (pH 7.4) containing 0 or 10 mM dithiothreitol (DTT, a reductive stimulus agent) at a final CPT equivalent concentration of 10 μg/ml. The kinetic change in the CPT fluorescence was recorded to evaluate CPT release. As shown in Figure 3(E), the image of CPT-SS-CUR/CM immediately changed from faint yellow to blue (within 3 min) when observed under a UV lamp with excitation at 365 nm. For CPT-CUR/CM, at least 8 h of incubation was required for an obvious change in the appearance under the same condition. This result was confirmed by the kinetic changes of emission spectra: the intensity of CPT fluorescence of CPT-SS-CUR/CM remarkably increased upon incubation, whereas no obvious changes in the emission spectra were detected within 4 h for CPT-CUR/CM. These data indicated that CPT-SS-CUR/CM showed a high reductive-responsiveness to release CPT as compared with that of CPT-CUR/OA. In absence of DTT, both CPT-SS-CUR/CM and CPT-CUR/CM displayed no remarkable changes in the emission spectra (Figure 3(F)), which suggested that both CPT-OA and CPT-SS-OA were stable upon incubation in 10 mM PB (pH 7.4).

CPT release from CPT-OA/CM and CPT-SS-OA/CM were also investigated using high performance liquid chromatography (HPLC). As shown in Figure 3(G), there is nearly 100% CPT released from the CPT-SS-OA/CM within 1 h upon incubation in 10 mM DTT. By contrast, nearly no CPT was released after 24 h for CPT-OA/CM under the same condition. Such strikingly different release behavior was ascribed to the presence of disulfide bond in CPT-SS-OA, endowing it with the ability to selectively release CPT once triggered by a reductive stimulus. Furthermore, it is shown that the concentration of close-ring CPT was higher than that of open-ring CPT within the initial 1 h (Figure 3(H)). Considering that the majority of CPT existed in the open-ring form at pH 7.4 (around 90%) (Martino et al., 2017), this result suggested that the CPT was mainly released from CPT-SS-OA/CM in the form of active lactone CPT rather than the inactive carboxylate CPT.

### 3.3. *In vitro* cytotoxicity and cellular uptake

**Figure 4.**
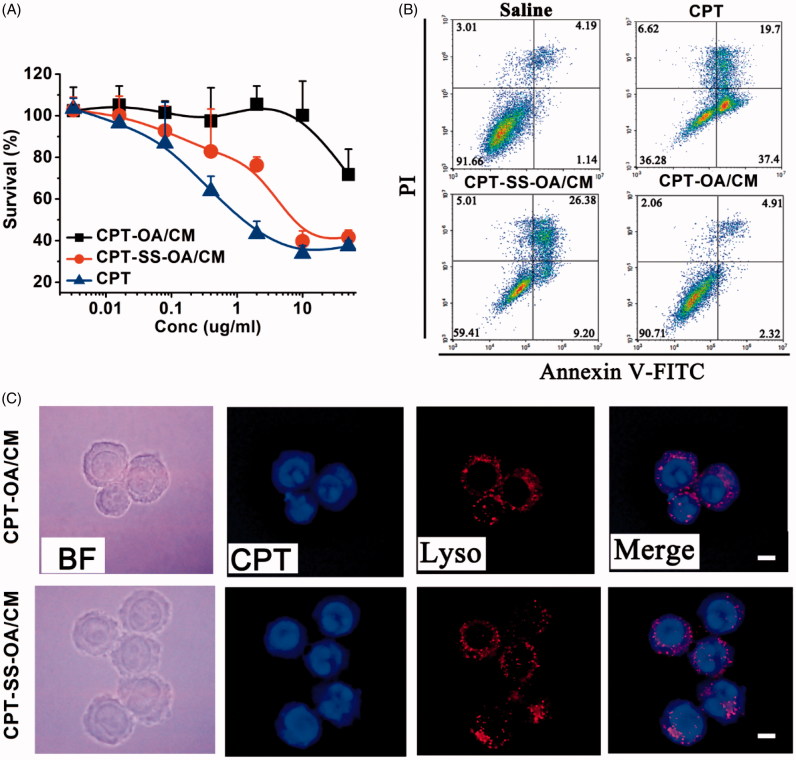
Comparative studies on CPT-OA/CM and CPT-SS-OA/CM: cytotoxicity study against the LLC cells determined by MTT assay (mean ± SD, *n* = 4) (A). Apoptotic analysis of LLC cells using an Alexa Fluor 488 Annexin V/PI Detection Kit after 48 h of incubation at a CPT equivalent concentration of 1 μg/ml (B). Fluorescence microscopy images of LLC cells cultured with CPT-OA/CM and CPT-SS-OA/CM at a CPT equivalent concentration of 5 μg/ml for 2 h (scale bar, 10 μm) (C).

*In vitro* cytotoxicity of CPT-SS-OA/CM and CPT-OA/CM were evaluated in LLC cells using MTT assay. As shown in Figure 4(A), CUR-OA/CM exhibited nearly no cytotoxicity against LLC cells within the studied range of CPT concentrations. By contrast, CUR-SS-OA/CM showed a much more potent cytotoxicity than that of CUR-OA/CM. To further compare their anti-proliferative effect, the fluorescence-activated cell sorting (FACS) analysis was used to detect the cells apoptosis after incubation with CUR-SS-OA/CM and CUR-OA/CM at an equivalent CPT concentration of 1 μg/ml for 48 h at 37 °C. As shown in Figure 4(B), CUR-SS-OA/CM displayed the higher apoptosis as compared with that of CUR-SS-OA/CM (35.58% versus 7.23%). These results indicated that the presence of disulfide bond in LHPs could significantly increase *in vitro* cytotoxicity, probably due to the accelerated drug release triggered by the intracellular elevated reductive GSH.

We next investigated the cellular uptake of CPT-SS-OA/CM and CPT-OA/CM in LLC cells using a fluorescence microscope. LLC cells were incubated with CPT-SS-OA/CM and CPT-OA/CM at a CPT equivalent concentration of 5 μg/ml for 2 h at 37 °C before imaging. As shown in Figure 4(C), CPT signal in blue was not strictly localized with lysosomal signal in red, and the extensive blue fluorescence outside the lysosome was observed. Interestingly, a clear CPT fluorescence was found in the cytomembrane, probably due to the insertion of CPT-OA into cell membranes. These results suggested that both CPT-SS-OA and CPT-OA entered LLC cells mainly through a direct transmembrane transportation rather than a lysosome-dependent pathway.

### 3.4. Pharmacokinetics

**Figure 5.**
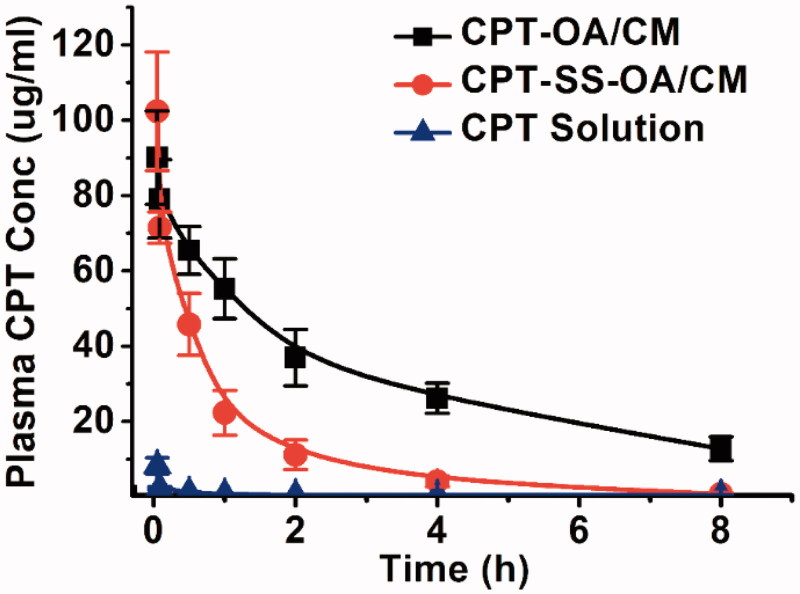
Plasma concentration–time profiles in rat following a single intravenous administration of CPT-OA/CM, CPT-SS-OA/CM and CPT solution at a CPT equivalent dose of 3 mg/kg (mean ± SD, *n* = 4).

Pharmacokinetics was studied in male Sprague-Dawley rats. CPT-OA/CM, CPT-SS-OA/CM and CPT solution were intravenously injected at an equivalent CPT dose of 3 mg/kg. As shown in Figure 5, free CPT was rapidly cleared from blood (AUC, 4.7 μg/ml·h), which was in good agreement with the previous reports (Tang et al., 2014). The plasma AUC for CPT-OA/CM and CPT-SS-OA/CM was 253.3 and 86.3 μg/ml·h, which was around 53.9 and 18.4 times of that of CPT solution (*p* < .001), respectively. This result indicated that lipophilic CPT prodrug-loaded CM showed a much longer circulating time than that of CPT solution, which provided a chance enabling more CM-encapsulated prodrugs accumulated into tumor via the enhanced permeation and retention (EPR) effect (Fang et al., 2011). These results agreed well with the previous reports, in which DHA-PTX formulated in CM exhibited a remarkably higher plasma AUC than that of Taxol both in mice and human (Bradley et al., 2001b; Wolff et al., 2003). It is worth mentioning that CPT-OA/CM displayed significantly prolonged blood circulation as compared to CPT-SS-OA/CM (*p* < .001), which was ascribed to the poorer chemical stability of CPT-SS-OA as compared with that of CPT-OA (Li et al., 2018b).

### 3.5. Biodistribution

**Figure 6.**
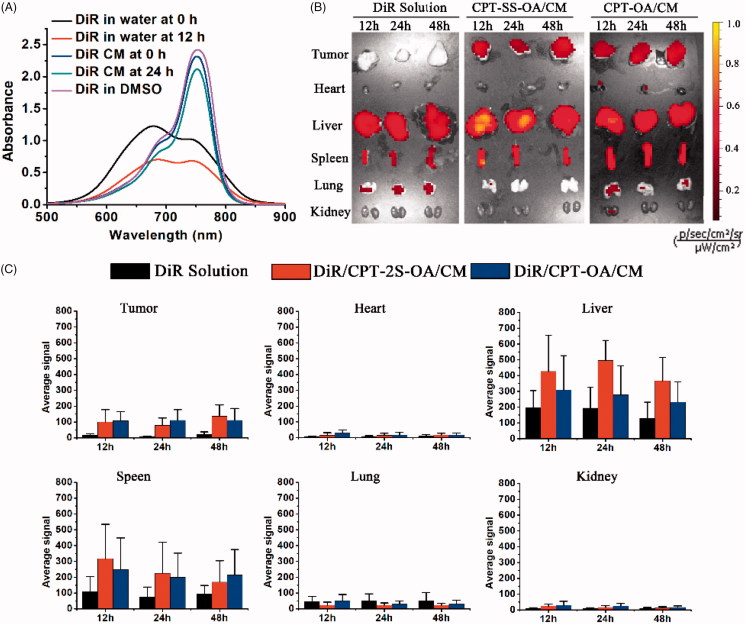
UV–Vis spectra of DiR-labeled CM, DiR solution in water and DMSO at 20 μg/ml before and after incubation at 37 °C for 12 h (A). *In vivo* distribution of DiR-labeled CPT-OA/CM, CPT-SS-OA/CM in the subcutaneous CT26 tumor-bearing mice post intravenous injection at an equivalent CPT dose of 10 mg/kg: fluorescence imaging (B) and quantitative analysis (C) of organs and tumor at 12, 24, and 48 h (*n* = 1).

DiR was used to label CPT-OA/CM and CPT-SS-OA/CM for the biodistribution study, and the DiR retention in CM was evaluated by detecting its absorption spectrum upon incubation. As shown in Figure 6(A), DiR-labeled CM showed the absorption spectrum similar to that of DMSO solution of DiR (existing in monomeric species), whereas the DiR in water exhibited a remarkably different UV–Vis spectrum. This result indicated that the CM-entrapped DiR was in a less aggregated stage as compared with that in water. Moreover, there was no significant change in the UV-vis spectrum of DiR-CM after 12 h incubation at 37 °C. This indicated that DiR could be stably encapsulated in CM, which probably arose from the co-presence of long alkyl chain both in Cremophor EL and DiR.

We next investigated the biodistribution of CPT-OA/CM and CPT-SS-OA/CM in the CT26 tumor-bearing BALB/C mice using the *ex vivo* fluorescence imaging. As shown in Figure 6(B), DiR-labeled CPT-OA/CM and CPT-SS-OA/CM showed a strong fluorescent signal in tumors at various time points as compared with that of free DiR. Quantitative analysis indicated that both CPT-OA/CM and CPT-SS-OA/CM showed 5–13 folds higher fluorescence intensities in tumor tissues than that of free DiR (Figure 6(C)). These results suggested that the lipophilic prodrugs in CM could successfully accumulate in tumors via the EPR effect (Fang et al., 2011). This result indicated that the higher tumor accumulation of the previously reported CrEL-formulated DHA-PTX might be ascribed, at least in part, to their passive accumulations in tumors via the EPR effect (Bradley et al., 2001a; Wolff et al., 2003).

### 3.6. Antitumor activity study in LLC tumor-bearing C57 mice

**Figure 7.**
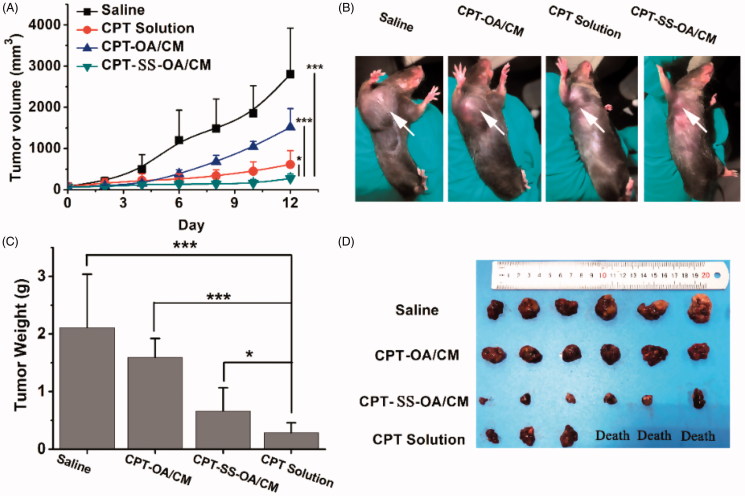
*In vivo* antitumor activity of CPT-OA/CM and CPT-SS-OA/CM against subcutaneous LLC tumor in C57 mice (*n* = 6) at an equivalent CPT dose of 10 mg/kg (every 3 days × 3): tumor growth curves (A); pictures of tumors after last treatment (B); weight (C) and images (D) of dissected tumors, *p < .05, ***p < .001.

LLC tumor-bearing C57 mice were used to investigate the *in vivo* antitumor effect of CPT-OA/CM and CPT-SS-OA/CM. As the unmodified CPT has a very low aqueous solubility, the water-soluble open-ring CPT obtained through lactone ring hydrolysis was applied as the positive control in this study. Although the carboxylate form of CPT is inactive, it can be gradually converted into the active closed-ring CPT after intravenous administration with a conversion degree of around 10% (Martino et al., 2017). As shown in Figure 7, CPT solution showed a moderate antitumor effect with the delayed tumor progression as compared with saline groups (inhibition rate, 68.9%), but the treatment of CPT solution also resulted in the death of half the number of mice, indicating a severe toxicity. The CPT-OA/CM displayed a very poor antitumor activity, and no significant difference was found when compared with the untreated control group after the last treatment (*p* = .113). In contrast, CPT-SS-OA/CM displayed significantly delayed tumor progression, with a tumor inhibition rate as high as 86.5%. The significantly improved antitumor effects of CPT-SS-OA/CM could be attributed to the accelerated CPT release from prodrugs as compared with CPT-OA/CM. Besides this, there are also other possible explanations accounting for the improved *in vivo* antitumor activity for the treatment of CPT-SS-OA/CM: (a) prolonged blood circulation, enabling the passive accumulation in tumors. (b) efficient cellular uptake through the transmembrane transportation of lipophilic CPT prodrugs. (c) GSH-triggered rapid and selective release of active lactone CPT in the tumor cells. CPT-SS-OA/CM undergoing these crucial delivery steps could finally delivery the lactone-stabilized CPT into tumor cells to achieve the improved chemotherapeutic efficacy.

**Figure 8.**
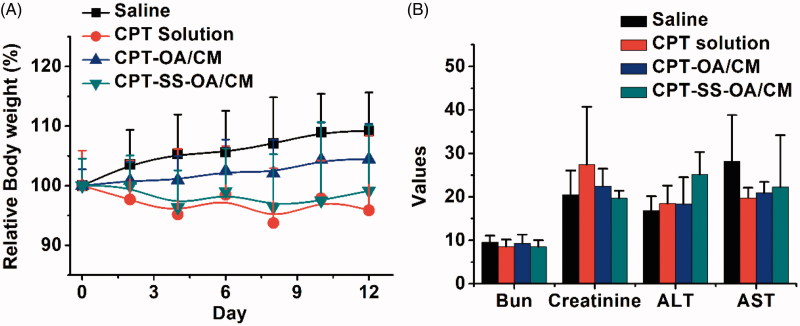
Body weight changes (A) and hepatic and renal function indicators (B) of LLC tumor-bearing C57 mice after treatment (*n* = 6).

Although there was no significantly difference in the average change of body weight between different treatment groups (Figure 8(A)), three mice in the CPT solution group showed a sharply decreased body weight and ultimately died with ∼20% weight loss, indicating an obvious toxicity of free CPT. In contrast, CPT-SS-OA/CM displayed a significantly attenuated toxicity (maximal loss of body weight, ∼10%) as compared with that of CPT solution, and no mice died from the CPT-induced toxicity after the last treatment. In comparison with CPT-SS-OA/CM, CPT-OA/CM exhibited a further decreased toxicity, with no loss in body weight observed for the CPT-OA/CM-treated mice. This result suggested that the high antitumor activity of CPT-SS-OA/CM was also associated with an enhanced toxicity as compared with CPT-OA/CM, which probably arose from its relatively poor chemical stability, enabling more CPT released from prodrugs during blood circulation. Additionally, there was no significant change in the indicators of hepatic and renal function observed in all the mice (Figure 8(B)), indicating the absence of the nephrotoxicity and hepatotoxicity.

**Figure 9.**
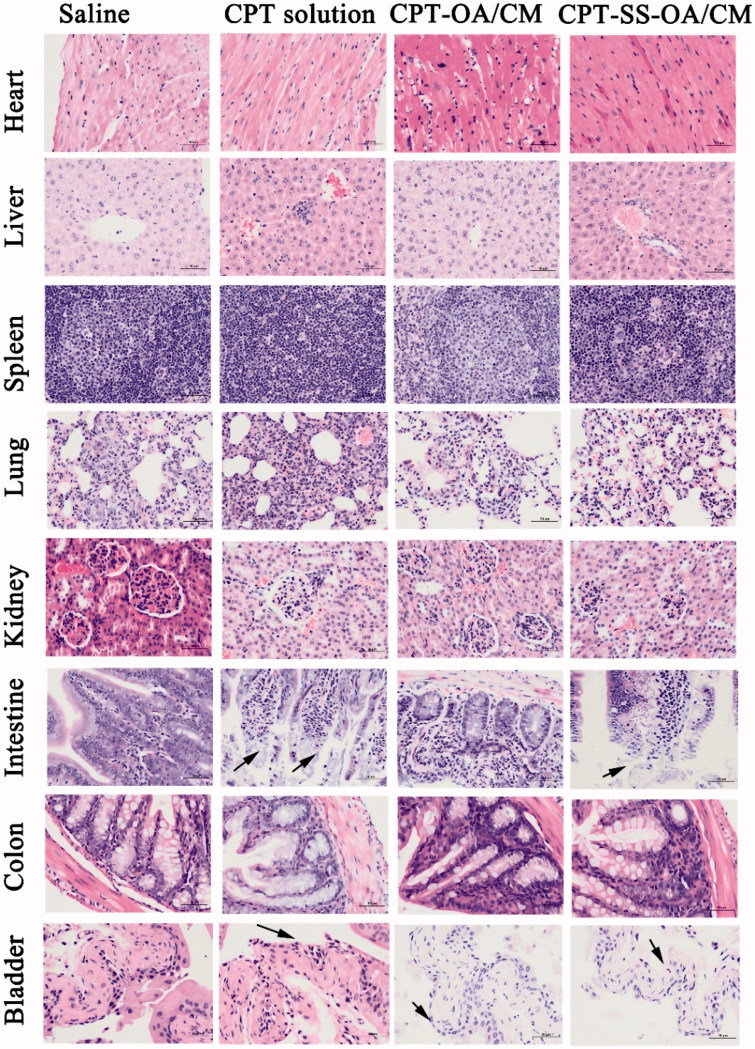
Histological section of vital organs (heart, liver, spleen, lung, kidneys, intestine, colon and bladder) stained with hematoxylin and eosin after intravenous treatment; autolytic changes in the intestine and epithelium loss in the bladder were marked with blank arrows.

Histological examination of hematoxylin and eosin-stained tissue section indicated that there were no significant histologic changes observed in heart, liver, spleen, lung, kidneys, and colon, indicating the absence of tissue damage in these organs (Figure 9). However, there were autolytic changes in the intestinal villi and the loss of bladder epithelium for mice in the CPT solution treated group, indicating obvious tissue damages in the intestine and bladder (Schmid et al. 2014). Such morphological changes (with a less degree) were also observed in the treatment group of CPT-SS-OA/CM, indicating that redox-sensitive lipophilic prodrugs of CPT could not fully eliminate CPT-induced toxicity. The intestinal toxicity was expected for CPT solution and CPT-SS-OA/CM, which caused the loss of body weight of mice, especially for the treatment of CPT solution that resulted in the death of half the number of mice. Although CPT-SS-OA/CM showed a higher toxicity than that of CPT-OA/CM, it still achieved an attenuated toxicity as compared with that of CPT solution. In comparison with CPT-SS-OA/CM, non-sensitive CPT-OA/CM induced lesser degree of histological change in the intestine and bladder, which could be ascribed to the slow release rate of CPT after administration. These data indicated that only CPT-SS-OA/CM showed an improved antitumor efficiency in the LLC cancer xenograft, despite both CPT-SS-OA/CM and CPT-OA/CM displayed several delivery advantages (i.e. prolonged blood circulation, tumor accumulation via EPR effect and efficient cellular uptake).

## 4. Conclusion

A reduction-sensitive lipophilic prodrug of CPT (CPT-SS-OA, containing disulfide bond) was used to deliver the lactone-stabilized CPT into tumor cells for the improved chemotherapeutic efficacy. To illuminate the role of disulfide bond, the hexyl carbonate-linked CPT-OA was used as the non-sensitive control. *In vitro* studies indicated that CPT-SS-OA/CM was readily internalized into cell and consequently converted to active lactone CPT, resulting in a higher cytotoxicity than that of CPT-OA/CM. Although both CPT-SS-OA/CM and CPT-OA/CM displayed a prolonged blood circulation time and tumor accumulation, only CPT-SS-OA/CM showed an improved antitumor efficiency in the LLC lung cancer xenograft as compared to CPT solution. These results indicated that redox-sensitive lipophilic conjugates may represent an effective approach for the delivery of active closed-ring CPT for improved antitumor efficacy, and such strategy can also be applied to other hydrophobic drugs with poor chemical stability.
